# Impact of Blood–Brain Barrier to Delivering a Vascular-Disrupting Agent: Predictive Role of Multiparametric MRI in Rodent Craniofacial Metastasis Models

**DOI:** 10.3390/cancers14235826

**Published:** 2022-11-26

**Authors:** Shuncong Wang, Yuanbo Feng, Lei Chen, Jie Yu, Yue Li, Yicheng Ni

**Affiliations:** 1 KU Leuven, Biomedical Group, Campus Gasthuisberg, 3000 Leuven, Belgium; 2 Shanghai Key Laboratory of Molecular Imaging, Shanghai University of Medicine and Health Sciences, Shanghai 201318, China

**Keywords:** brain metastasis, VDA, MRI, rodent, IHC, fosbretabulin

## Abstract

**Simple Summary:**

The role of the blood–brain barrier in intracranial cancer treatment by vascular-disrupting agents remains unclear. Intraindividual comparison of vascular shutdown by a craniofacial metastasis model was performed, on the basis of in vivo MRI imaging, postmortem NanoCT and pathology analyses. The differential VDA therapeutic effects between intracranial and extracranial tumors were observed, with stronger vascular shutdown in the extracranial tumor. In vivo multiparametric MRI may serve as a predictor for the efficacy of VDAs on craniofacial tumors.

**Abstract:**

Vascular-disrupting agents (VDAs) have shown a preliminary anti-cancer effect in extracranial tumors; however, the therapeutic potential of VDAs in intracranial metastatic lesions remains unclear. Simultaneous intracranial and extracranial tumors were induced by the implantation of rhabdomyosarcoma in 15 WAG/Rij rats. Pre-treatment characterizations were performed at a 3.0 T clinical magnet including a T2 relaxation map, T1 relaxation map, diffusion-weighted imaging (DWI), and perfusion-weighted imaging (PWI). Shortly afterward, a VDA was intravenously given and MRI scans at 1 h, 8 h, and 24 h after treatment were performed. In vivo findings were further confirmed by postmortem angiography and histopathology staining with H&E, Ki67, and CD31. Before VDA treatment, better perfusion (AUC30: 0.067 vs. 0.058, *p* < 0.05) and AUC300 value (0.193 vs. 0.063, *p* < 0.001) were observed in extracranial lesions, compared with intracranial lesions. After VDA treatment, more significant and persistent perfusion deficiency measured by PWI (AUC30: 0.067 vs. 0.008, *p* < 0.0001) and a T1 map (T1 ratio: 0.429 vs. 0.587, *p* < 0.05) were observed in extracranial tumors, in contrast to the intracranial tumor (AUC30: 0.058 vs. 0.049, *p* > 0.05, T1 ratio: 0.497 vs. 0.625, *p* < 0.05). Additionally, significant changes in the T2 value and apparent diffusion coefficient (ADC) value were observed in extracranial lesions, instead of intracranial lesions. Postmortem angiography and pathology showed a significantly larger H&E-stained area of necrosis (86.2% vs. 18.3%, *p* < 0.0001), lower CD31 level (42.7% vs. 54.3%, *p* < 0.05), and lower Ki67 level (12.2% vs. 32.3%, *p* < 0.01) in extracranial tumors, compared with intracranial lesions. The BBB functioned as a barrier against the delivery of VDA into intracranial tumors and multiparametric MRI may predict the efficacy of VDAs on craniofacial tumors.

## 1. Introduction

Brain metastasis (BM) is a common complication in patients with malignant tumors, especially non-small-cell lung cancer, melanoma, and breast cancer [1]. It is estimated that 20–30% of cancer patients may develop BM; however, the exact epidemiological data are not reported yet because of the possible under-estimated incidence due to (1) lack of mandatory reporting of BM status in all patients; and (2) lack of routine imaging surveillance of brain during clinical management [2]. This figure may likely increase with time, due to prolonged patient survival and increased sensitivity in detecting BM thanks to the technical development of imaging modalities.

BM represents an epidemiologically distinct metastasis, compared with liver and lung metastasis, for the same primary tumor [3,4,5]. Due to the unique microenvironment of the brain, i.e., dense cellularity, blood–brain barrier (BBB), and cerebrospinal fluid (CSF), therapeutics that are effective to control primary disease are usually futile to eliminate cancer cells disseminated into the brain. This can be attributed to two major factors: (1) cancer cell evolution and/or selection (harboring novel mutations or metabolic profiling during metastasis [6]; and (2) BBB-limited drug penetration and/or tumor retention [7]. Currently available therapeutics for BM include ALK, EGFR-targeting therapies for lung cancer, for breast cancer with HER2 and for melanoma with BRAF gene, but they consist of only 18% BM cases in total [8,9]. For other BM patients without known druggable targets, immune checkpoint inhibitors showed encouraging cancer control in melanoma, triple-negative breast cancer, and NSCLC-derived BM [10]. Besides focusing on the already-existing driving mutation in primary lesions, additional mutations having developed during metastasis may be another potential target, e.g., FAM129C and ADAMTSs in BM lesions but absent in primary lung cancer [11].

The BBB is constructed of a continuous layer of capillary endothelial cells connected by inter-cellular tight junctions and adherent junctions, a basement membrane, pericytes, and end-foot processes of perivascular astrocytes, whereas in the site of BM, the BBB is compromised with heterogeneous drug permeability [12].

Besides strategies focusing on cancer cells, tumor stromal cells may serve as an alternative target, which are less diverse than mutative cancer cells and more homogenous among different cancer types. Uncontrollable growth, invasive capacity, and metastatic invasion into distant organs or tissues are major hallmarks of malignancies, all of which highly depend on the proliferating vascular networks [13]. Thus, targeting tumoral blood vessels has a great implication for controlling cancers by either inhibition of neoangiogenesis or disruption of existing blood vessels, resulting in tumor starvation and consequently necrosis. The former type represented by Bevacizumab (Avastin^®^) targets angiogenesis and have shown survival benefit to glioblastoma multiforme in a recurrent setting [14], but not in a first-line setting [15]. Thus, the latter group of anti-vascular agents includes vascular-disrupting agents (VDAs) as typically exemplified by combretastatin A-4 phosphate (CA4P), which have shown preliminary therapeutic effects in liver cancer and lung cancer [16,17,18]. However, the limited efficacy of VDAs on an orthotopic glioma model has been shown [19]. However, it would be interesting to question to what extent the existence of the BBB may impact the therapeutic effect of a VDA on BM as compared to extracranial tumors. Furthermore, exploring the efficacy of VDAs in intra- and extracranial tumors may facilitate our ongoing study on the dual-targeting pan-anticancer strategy OncoCiDia for brain tumors [20,21,22].

Given the dynamically altering genetic profiles in cancer cells, choosing a treatment modality that only targets tumor stromal cells deems to be more clinically relevant for a comparison between intracranial and extracranial settings. CA4P, fosbretabulin, is a classical VDA derived originally from African bush willow Combretum caffrum, which binds to tubulin and destroys the cytoskeleton in the endothelial cells of tumor blood vessels. Thus, based on orthotopic craniofacial tumor models of immunocompetent rodents [23], the present intraindividual comparative study was designed to test the follwoing working hypotheses: (1) a VDA may impose diverse efficacies between intracranial and extracranial tumors with the same origin but different locations; and (2) pre-treatment MRI may provide suggestive information for therapeutic prediction and patient selection.

## 2. Materials and Methods

### 2.1. Study Design

**Figure 1:**
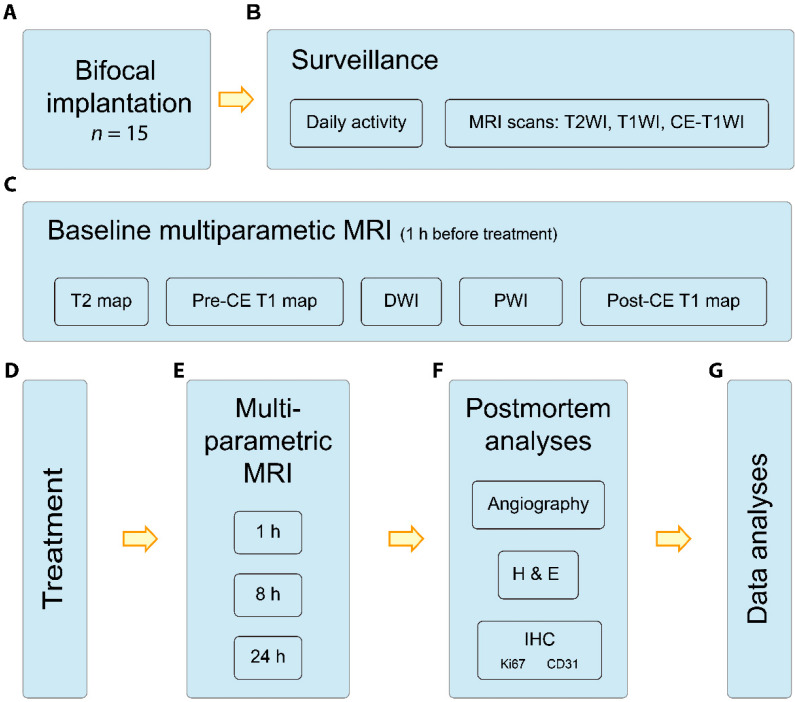
Flow chart for current study. Flow chart for comparison of therapeutic effect of VDA on intracranial and extracranial tumors; briefly, tumor implantation (**A**), follow-up of status and tumor growth on 3.0 T MRI (**B**), multiparametric characterization of BM lesions (**C**), delivery of VDA (**D**), post-treatment MRI surveillance (**E**), postmortem analyses after last MRI scan (**F**), and data analyses (**G**). Abbreviations: MRI: magnetic resonance imaging, T2WI: T2-weighted imaging, T1WI: T1-weighted imaging, CE-T1WI: contrast-enhanced T1-weighted imaging, PWI: perfusion-weighted imaging, T2map: T2 relaxation map, pre-CE T1map: pre-contrast-enhanced T1 relaxation map, post-CE T1map: post-contrast-enhanced T1 relaxation map, CE: contrast-enhanced; H&E: hematoxylin and eosin stain, IHC: immunohistochemistry staining, CD31: cluster of differentiation 31, and Ki67: a nuclear protein marker for proliferating cells.

As shown in Figure 1, this study was executed in line with standard animal care after approval by the ethical committee of KU Leuven (No. P046/2019). A literature search was performed to gain an overview of previous publications, with special emphasis on the (1) orthotopic intracranial tumor; and (2) application of VDAs (Supplementary Figure S1A, Supplementary File S1). 

### 2.2. Simultaneous Intracranial and Extracranial Tumor Model

Sample size was defined empirically and based on the nature of intraindividual comparison in this study. Anaesthetization was given with 2% isoflurane in a mixture of 20% oxygen and 80% air (Harvard Apparatus system, Holliston, MA, USA) for both surgery and in vivo imaging. Fifteen WAG/Rij rats (eight male and seven female) of 11 weeks of age (Charles River Breeding Laboratories, Inc., Elbeuf, France) were surgically implanted with 1.0 mm^3^ cubic rhabdomyosarcoma tissue. Intracranial and extracranial tumors were induced simultaneously, with the intracranial tumor based on a previously published methodology (Figure 1A) [23]. Extracranial tumor lesions were also created in the facial muscle at a cross-sectional level similar to that of the intracranial ones for direct comparison of lesions in the same slice of the MRI scan. Post-surgically, these animals were housed in an animal facility under close and daily observation of vital signs, activity, neurological status, social interaction, and food ingestion. Regular anatomical MRI surveillance started one week after the implantation every two days at a 3.0 T scanner (MAGNETOM Prisma; Siemens, Erlangen, Germany) with a 16-channel phase array wrist coil (Figure 1B). Euthanasia was executed shortly after the last MRI scans or whenever the rats reached the humane endpoints during the experiment [24]. The humane endpoints adopted specifically in this study included tumor size (largest dimension > 7 mm), behavior abnormality, and weakness.

### 2.3. VDA Treatment

Whenever the largest dimension of the intracranial tumor growth reached 4 mm (around 14 days after implantation) as detected by surveillance MRI (Figure 1C), CA4P (C643025, Toronto Research Chemical, Inc. Toronto, Canada) was dissolved in 0.9% normal saline and intravenously (iv) infused through a lateral tail vein at a dose of 20 mg/kg during 5 min. As a classical VDA, CA4P is a small molecular disodium phosphate hydrophilic prodrug of combretastatin A-4 (CA4) (Figure 1D, Supplementary Figure S1B). The therapeutic effect of VDA on either lesion was illustrated by comparing with pre-treatment by perfusion-related MRI sequences as described below.

### 2.4. MRI Acquisitions

One hour prior to the above VDA treatment, a baseline MRI was performed to characterize the tumor status, including anatomical scans by 3D T2WI, 3D T1WI and 3D CE T1WI, and multiparametric scans of T2 mapping, T1 mapping (without/with contrast agent), diffusion-weighted imaging (DWI), and perfusion-weighted imaging (PWI). Multiparametric MRI was repeated at 1, 8, and 24 h post-treatment (Figure 1E). All the MRI sequences here were adopted from the clinically used ones after proper optimization. Specifically, the T2 value was estimated with 24 different echo times and the T1 value was approximated using the variable flip angle method with 9 different flip angles [23]. For the PWI, a gadolinium-based contrast agent (CA) Dotarem^®^ (Gd-DOTA, Guerbet, Roissy CdG Cedex, France) at 0.1 mmol/kg was used in the dynamic contrast enhancement setting, with 20 frames of pre-contrast, 80 frames of post-contrast scans, and a temporal resolution of 3.6 s [25,26] (Figure 1F,G).

### 2.5. Postmortem NanoCT Scan and Pathology

Rats were euthanized by an intraperitoneal overdose of pentobarbital (Nembutal; Sanofi Sante Animale, Brussels, Belgium) after the last MRI scans. An amount of 1.5 mL of barium sulphate suspension (Guerbet, Villepinte, France) was injected via the aorta and used as a CA for NanoCT scans (Phoenix Nanotom M, GE Measurement and Control Solutions, Boston, MA, USA). Brain and extracranial tumor lesions were excised and fixed in 4% PBS-buffered formalin. NanoCT scans were performed to visualize blood vessels in tumors with a resolution of 15 μm. Brain and extracranial tumor specimens were sliced at a thickness of 8 μm for hematoxylin–eosin (H&E) staining to identify and delineate tumor necrosis, and for IHC staining of Ki67 (1:1200; Abcam, ab15580, Cambridge, UK), and CD31 (1:400; Abcam, ab182981, Cambridge, UK) to quantify cancer cell proliferation, and blood vessels, respectively (Figure 1G).

### 2.6. Image Analyses

MRI images were pre-processed and analysed after conversion into Nifti, with the region of interest (ROI) being semi-automatically captured by ITK-SNAP (v3.2.0, www.itksnap.org) and manually corrected by two radiologists (YB Feng and YC Ni) independently [27]. Both pixel-wise and ROI-based methods were adopted based on the mathematical equations (Supplementary File S2) [28]. Here, AUC30 was defined as the area under the CA concentration curve for the first 30 s since CA injection and used as a surrogate of perfusion. Similarly, AUC300, defined as the area under the CA concentration curve for the first 300 s since CA injection, was utilized as a measure of VDA perfusion plus retention. Contralateral brain tissue was used as a control (brain tissue).

NanoCT images were reconstructed after exportation as tiff files performed by the Dataviewer and CTvox (Bruker MicroCT, Bremen, Germany) and shown by video visualization. Positive rate, defined as the percentage of cells considered positively stained over all cells, of either Ki67 or CD31 was calculated under three randomly selected ×200 magnification field. Additionally, vascularity was assessed by (1) relative vascularity by NanoCT with contralateral brain tissue as reference; and (2) average area of blood vessel delineated by CD31 under microscopy.

### 2.7. Statistical Analyses

All statistical analyses in the study were performed in R (v3.1.1, https://cran.r-project.org, accessed on 1 January 2020) [29] (Figure 1G). The normalization test was performed using Shapiro–Wilk’s method, after which paired t-tests were adopted for intraindividual comparison [30]. The figures were made using GraphPad Prism (version 9.0.0 for Windows, GraphPad Software, USA, www.graphpad.com, accessed 1 January 2019). A two-sided *p* < 0.05 was considered as statistically significant.

## 3. Results

### 3.1. General Aspects of the Study

**Figure 2:**
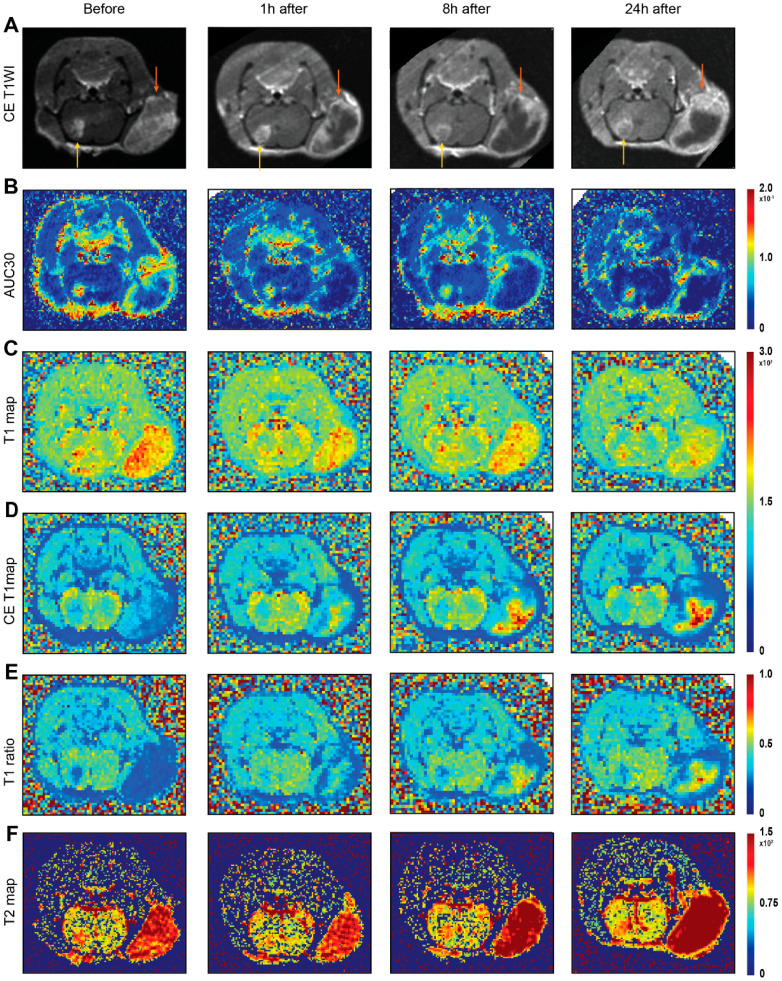
Exemplified case of intraindividual comparison of intracranial and extracranial tumors before and after VDA treatment. CE-T1WI anatomical scans for cases before treatment, 1 h, 8 h, and 24 h (**A**) after VDA treatment, AUC30 (**B**), T1 relaxation map (**C**), CE T1 relaxation map (**D**), T1 ratio map (**E**) and T2 relaxation map (**F**) before treatment, 1 h, 8 h, and 24 h after CA4P treatment. Intracranial and extracranial lesions are indicated by yellow and orange arrows, respectively. Abbreviations: VDA: vascular-disrupting agent, CE: contrast enhancement, AUC30: area under the curve for the first 30 s.

The literature search identified a relevant study elaborating on the differential anti-cancer efficacy of VDA (DMXAA) between intracranial and extracranial settings based on a glioma mice model [31] (Supplementary Figure S1A, Supplementary File S1). However, no comparison in a brain metastasis setting was reported. All rats survived and tolerated well the experimental procedures including anesthesia, tumor implantation, VDA administration, and serial MRI scanning without observable abnormality until the designated endpoint of 24 h after CA4P treatment and euthanasia after the last MRI exam. Despite the same tumor tissue implanted with the same method on the same day, intracranial tumors grew more slowly than extracranial ones did, resulting in an over 5-fold volumetric difference (Figure 2). MRI acquisitions at a 3.0 T clinical magnet were successfully achieved for both structural and functional assessments of rodent craniofacial metastasis models with intensive computer-assisted multiparametric imaging analyses. The applied postmortem 3D nano-CT microangiography helped verify in vivo MRI findings on tumor responses to VDA.

### 3.2. Pre-Treatment MRI Characterization of Tumors

**Figure 3:**
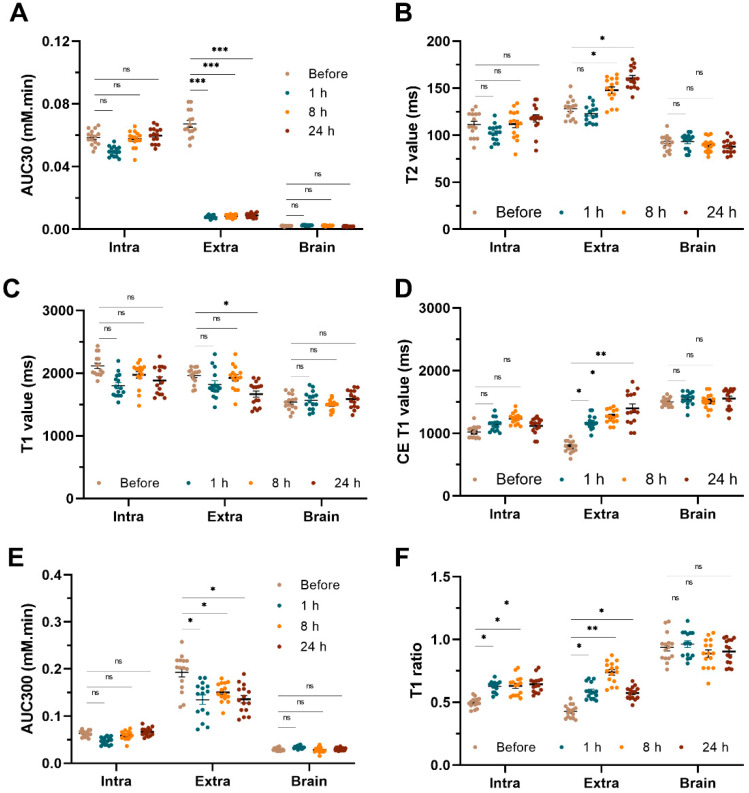
Statistical analyses of multiparametric MRI. Statistical analyses of AUC30 (**A**), T2 value (**B**), T1 value (**C**), CE T1 value (**D**), AUC300 value (**E**) and T1 ratio (**F**) before VDA treatment, 1 h, 8 h, and 24 h after VDA treatment. Abbreviations: AUC30: area under the curve for the first 30 s, VDA: vascular-disrupting agent. ns: non-significant, *: *p* < 0.05, **: *p* < 0.01, ***: *p* < 0.001.

Before VDA treatment, better perfusion was observed in extracranial lesions, as indicated by the AUC30 (0.067 vs. 0.058, *p* < 0.05) and the lower contrast-enhanced (CE) T1 value (786.1 vs. 1020.9, *p* < 0.01) (Figure 2B,E and Figure 3A,D, Supplementary Figure S4A–D, Supplementary Video S1). Additionally, extracranial lesions showed a higher T2 value (128.2 vs. 111.5, *p* < 0.05) and T1 value (Figure 2C,F and Figure 3B,C) compared with intracranial lesions. AUC300, the area under the curve of the first five minutes in PWI, showed significantly higher concentrations of contrast agent accumulating in extracranial lesions, indicating more drug accumulation there (0.193 vs. 0.063, *p* < 0.001) (Supplementary Figure S2B and Figure 3E). 

### 3.3. Differential Responses towards VDA Treatment

At one hour after CA4P treatment, all extracranial lesions showed significant perfusion deficiency as measured by AUC30, compared with the slight perfusion deficiency in the intracranial lesions (0.008 vs. 0.049, *p* < 0.001) (Figure 3A). At eight hours after treatment, VDA-related perfusion deficiency persisted in extracranial lesions, compared with intracranial lesions where the perfusion was nearly restored, as shown by AUC30 (Figure 2). At 24 h after treatment, slight restoration of VDA-related perfusion deficiency could be observed in extracranial lesions especially at the tumor periphery, as shown by the higher AUC30 in periphery (Figure 2A). Other perfusion-related parameters such as the CE-T1 value and T1 ratio confirmed the dynamic evolution of tumor perfusion over time, i.e., an increased CE-T1 value and T1 ratio were observed in 1, 8, and 24 h after treatment in extracranial tumors (Figure 2D,E and Figure 3D). Additionally, significant increases in the T2 value (*p* < 0.05) were observed in extracranial lesions at 8 and 24 h after treatment (Figure 2F and Figure 3B) accompanied by a higher ADC value of increased water diffusion (Supplementary Figure S2A). No significant changes in the AUC30, T2 value, and T1 value over the four time points could be observed in the contralateral brain (Figure 3). Dynamic perfusion changes by time points (Supplementary Figure S3) and by locations (Supplementary Figure S4) were also demonstrated.

### 3.4. Postmortem Angiography and Pathology

**Figure 4:**
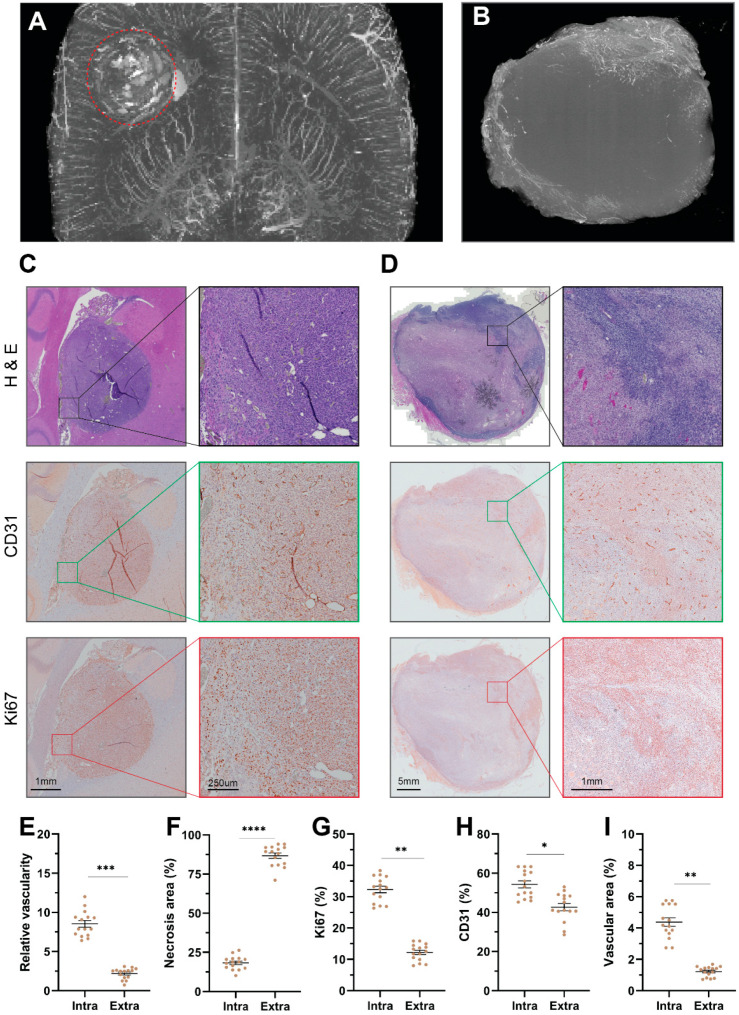
Nano-CT scans and pathological analyses. Nano-CT scans of intracranial tumor (**A**) and extracranial tumor (**B**). Exemplified cases receiving VDA of intracranial tumor (**C**), and extracranial tumor (**D**) after staining by H&E, CD31, and Ki67. Comparison of relative vascular density of brain tumor tissue compared with contralateral brain tissue (**E**), necrosis area (**F**), Ki67-positive rate (**G**), CD31-positive rate (**H**), and vascular area delineated by CD31 staining by pathology of either intracranial or extracranial tumor (**I**). Abbreviations: VDA: vascular-disrupting agent, CD31: cluster of differentiation 31 for vascular endothelia, Ki67: a nuclear protein marker for proliferating cells, *: *p* < 0.05, **: *p* < 0.01, ***: *p* < 0.001, ****: *p* < 0.0001.

As shown in microangiography, the intracranial lesion (red dotted circle) was fully re-perfused except for a small area of perfusion deficiency, with barium leakage (plaque-like signal) in the tumor periphery (Figure 4A, Supplementary Video S2). However, vascular shutdown was massively formed in the central extracranial lesion, with small areas of remnant perfusion in the tumor periphery (Figure 4B, Supplementary Video S2). Nano-CT quantification showed higher vascular volume in the intracranial lesions after treatment than that in the extracranial lesions (*p* < 0.001) (Figure 4E). These findings are in line with the in vivo AUC30 (Figure 2B), AUC 300 (Supplementary Figure S2B), and CE-T1 map (Figure 2D).

Angiographic findings were further confirmed by histopathological analyses. The postmortem pathology of HE and IHC staining techniques showed limited necrosis formation in intracranial lesions, in comparison with massive necrosis in the extracranial lesions (approximately 20% vs. 80%, *p* < 0.0001) (Figure 4C,D,F). Significantly lower Ki67 could be observed in extracranial lesions, indicated abrupted proliferation in extracranial lesions (Figure 4G). CD31 showed an interesting phenomenon: despite non-significant CD31-positive rates between intra- and extracranial tumors, a significantly lower (deflated) vascular area delineated by CD31 was observed only in extracranial lesions (Figure 4H,I).

## 4. Discussion

The current study demonstrated that (1) extracranial tumors showed better pre-treatment blood perfusion than intracranial tumors did, a phenomenon indicative of better drug penetration and/or distribution and, thus, therapeutic effect in favor of extracranial ones; (2) a better CA4P-induced vascular shutdown effect with a larger area of necrosis could be observed in the extracranial tumors, relative to intracranial lesions; and (3) such differential therapeutic efficacies could be reflected by a 3-fold concentration difference of CA Dotarem^®^ (thus, CA4P of a comparable molecular weight) as defined by AUC300. To our knowledge, this is the first intraindividually comparative study for multiparametric MRI analyses between intracranial and extracranial tumors, to explore the role of a disrupted BBB in intratumoral anti-cancer drug penetration and retention, in particular with CA4P as a representative VDA.

Here, we adopted dynamic 3D multiparametric MRI, instead of merely 2D dimensional morphology MRI, for longitudinal follow-up based on VDAs’ unique mechanism for inducing necrosis [32]. Striking difference between intra- and extracranial tumor lesions could be dynamically detected by DCE imaging and the contrast agent concentration could be further quantified by AUC30 and AUC300, from which pharmacokinetics of small molecular anti-cancer drugs with short plasma half-life such as VDAs could be extrapolated. These measures reflect a comprehensive presence of perfusion, diffusion, permeability, vascular volume, and microenvironment exemplified by disrupted BBB that interplay with a particular drug such as CA4P. These parameters may represent potential biomarkers to select patients who may benefit from VDA treatment. The endpoint defined at 24 h after VDA treatment for this study was due to the considered optimal addition to the patient on this time point with a necrosis-avid radioactive tracer for OncoCiDia strategy [17,18,19].

Despite the more homogeneous characteristics of tumor stroma, a different microenvironment also impacts the performance of VDAs, showing in this study with brain tumors as an extreme example. Intraindividual comparison between liver and pancreatic tumors of the same origin showed better response of liver cancer to CA4P, which could be attributed to the multiple arterial supplies to pancreatic tumors, which counteracted VDA’s vascular shutdown effect [33]. Similarly, an interindividual study comparing the therapeutic response to a different VDA DMXAA between ectopic (subcutaneous) and orthotopic (intramuscular) tumors showed a higher vascular volume in orthotopic tumors but a better vascular shutdown in ectopic tumors [34]. Another study on DMXAA comparing the therapeutic effect between intracranial and subcutaneous lesions indicated the marginal response in intracranial lesions [35].

The tumor microenvironment is crucial for angiogenesis through cross-talks between a network of tumor cells, stromal cells, and endothelial cells [36]. To better mimic the microenvironment, especially the presence of immune cells which are believed to be associated with angiogenesis, an immune-competent model was preferred [37]. The intra- and extracranial VDA efficacy in immune-compromised mice showed that the concentration, measured by LC–MS/MS, was 25-fold higher in subcutaneous tumors and, thus, more massive necrosis was observed [31]. However, the different perfusion characteristic between subcutaneous and orthotopic tumors was inconsistent: the orthotopic hormone-sensitive prostate tumor model showed poorer perfusion compared with subcutaneous tumors [38]. All the studies above, together with our findings, highlight the crucial role of the tumor microenvironment in stroma-targeting strategy. For further translational studies on especially perfusion-related therapy, pre-treatment MRI characterization could be predictive for cancer control and thus needed for the selection of patients who might benefit from VDAs.

Regarding the marginal therapeutic effect intracranially, there are two possibilities: (1) limited drug penetration or distribution; and (2) intrinsic unresponsiveness of the intracranial tumoral endothelium to VDAs. Based on the findings here, limited CA concentration in the brain caused by the BBB may help explain the poorer therapeutic effect. Further studies elaborating on the combination of focused ultrasound or other methods that help increase the local distribution or retention of anti-cancer drugs in the brain may better confirm the findings here. However, this is out of the scope of the current study. If the inferior therapeutic effect persists after fostered intracranial drug penetration and retention, another possibility would be the intrinsic unresponsiveness, which can be clarified from biological aspects via bioinformatics such as single-cell sequencing and metabolic profiling.

The BBB is a barrier maintaining brain physiological hemostasis, by which transportation across the BBB by both transcellular and paracellular pathways are strictly controlled. Under certain circumstances such as stroke, Alzheimer’s disease, and multiple sclerosis, remodeling of the inter-endothelial protein complex may lead to BBB breakdown [39]. During carcinogenesis, the BBB is abrupted with leaky characteristics in the tumor area. Both BBBs comprise the vascular environment of homing tumor cells [40]. Despite an initially leaky BBB in the brain, the duration and magnitude for the BBB opening remain largely unknown. Furthermore, the initial leakiness may be insufficient for delivering the required drugs with high enough concentrations for cancer control. Further BBB opening can be achieved either physiologically or mechanically, and the major rationale is to transiently disrupt the BBB by decreasing the expression of TJ protein such as claudin-1, occluding, and tricellulin. Example measures include high osmotic pressure induced by intra-arterial mannitol [41], cereport (a bradykinin analog) [42], and borneol [43]. Mechanically, focused ultrasound together with microbubbles disrupts the BBB by reducing the expression of tight junction protein, resulting in a temporary opening [44]. Practically, properly optimized DCE-MRI can evaluate the subtle BBB leakage with moderate-to-excellent reproducibility and thus serves as a powerful tool for facilitating development of novel BBB-opening agents [45]. Besides the increasing influx of anti-cancer agents into BM, the inhibition of efflux from the BBB mediated by transporters encoded by the ATP-binding cassette gene family, such as P-glycoprotein, would also be an alternative method or a method that can be combined with [25]. These transporters are proven to be functional even when the BBB integrity is disrupted [46].

This study was subject to the following limitations. Firstly, due to limited intracranial space and lower resolution of DWI, the comparison of ADC derived from the 3.0 T clinical magnet (Supplementary Figure S2B) was impaired by the partial volume effect. Secondly, in our study, measuring the concertation of VDAs was impossible since (1) the biological half-life for CA4P is around 25 min, and degradation happens at 24 h after treatment and during sampling; and (2) the loss of blood during sampling may lead to measurement bias of the VDA concentration. Thirdly, the AUC300 adopted here as a surrogate for drug perfusion should be interpreted with caution, due to the different molecular weight, charge distribution, and so on.

## 5. Conclusions

In conclusion, the present study demonstrated the differential VDA therapeutic effects between intracranial and extracranial tumors, which may lay a foundation for designing clinical trials in the future. Multiparametric MRI, particularly quantitative PWI parameters, may serve as a predictor for the efficacy of VDAs on brain tumors.

## Data Availability

All data in this study are available upon reasonable request. Python codes are available upon reasonable request.

## Supplementary Materials

The following supporting information can be downloaded at: https://www.mdpi.com/article/10.3390/cancers14235826/s1, Figure S1: Result of literature search and chemical structure of CA4P. Figure S2: Exemplified case of intra-individual comparison of intracranial and extracranial tumors before and after VDA treatment. Figure S3: Contrast agent concentration curve of the brain (A), intracranial tumor (B) and extracranial tumor (C) before treatment, one hour, eight hours and 24 h after treatment. Figure S4: Contrast agent concentration curve by tumor site before treatment (A), one hour (B), eight hours (C) and 24 h (D) after treatment. File S1: Protocol for literature research. File S2: Formulas for imaging analyses. Video S1: Example of dynamic contrast-enhanced perfusion weighted imaging intracranial and extracranial malignancies one hour before VDA treatment (A), one hour after treatment (B), eight hours after treatment (C) and 24 h after treatment (D). Abbreviations: VDA: vascular disrupting agent. mM: millimolar. Video S2: Angiography of tumor-bearing brain (A) and extracranial tumor (B) of the case shown in supplementary Video S1 by microCT.
