# Supplementary material for: Impact of Blood–Brain Barrier to Delivering a Vascular-Disrupting Agent: Predictive Role of Multiparametric MRI in Rodent Craniofacial Metastasis Models
Source: Cancers (Basel). 2022 Nov 26;14(23):5826. doi: 10.3390/cancers14235826 (PMC9740057; doi:10.3390/cancers14235826)
Supplement: Supplementary file 1 [file cancers-14-05826-s001.zip › cancers-2017974-supplementary.pdf]

Supplementary Materials

Supplementary Video S1. Example of dynamic contrast-enhanced perfusion weighted imaging intracranial and extracranial malignancies one hour before VDA treatment (A), one hour after treatment (B), eight hours after treatment (C) and 24 hours after treatment (D). Abbreviations: VDA: vascular disrupting agent. mM: millimolar.

Supplementary Video S2. Angiography of tumor-bearing brain (A) and extracranial tumor (B) of the case shown in supplementary video 1 by microCT.

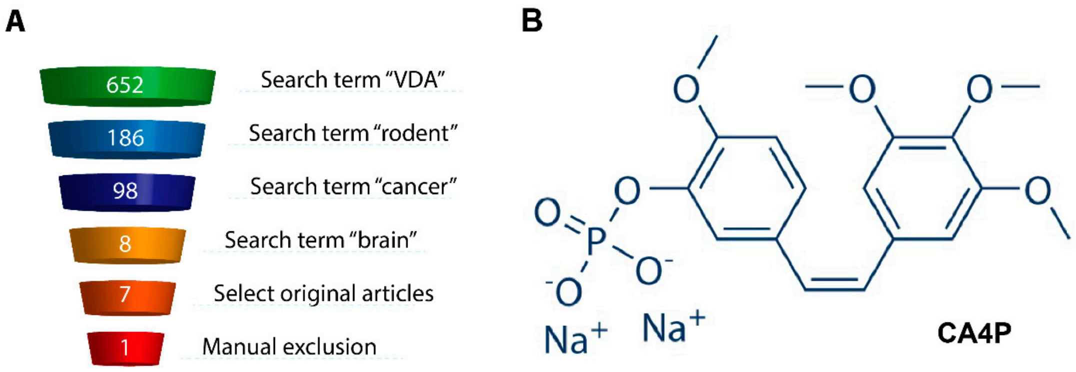

Figure S1. Result of literature search and chemical structure of CA4P.

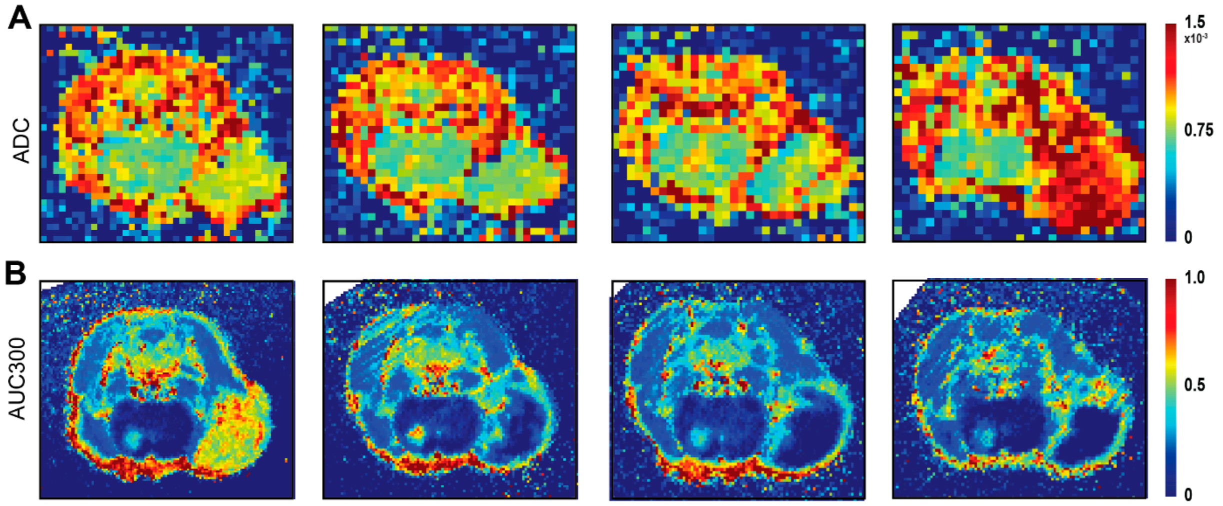

Figure S2. Exemplified case of intra-individual comparison of intracranial and extracranial tumors before and after VDA treatment. ADC map (A) and AUC300 map (B) for the case before treatment, one hour, eight hours and 24 hours after VDA treatment. Abbreviations: VDA: vascular disrupting agent, ADC: apparent diffusion coefficient, AUC300: area under curve for the first 300 seconds.

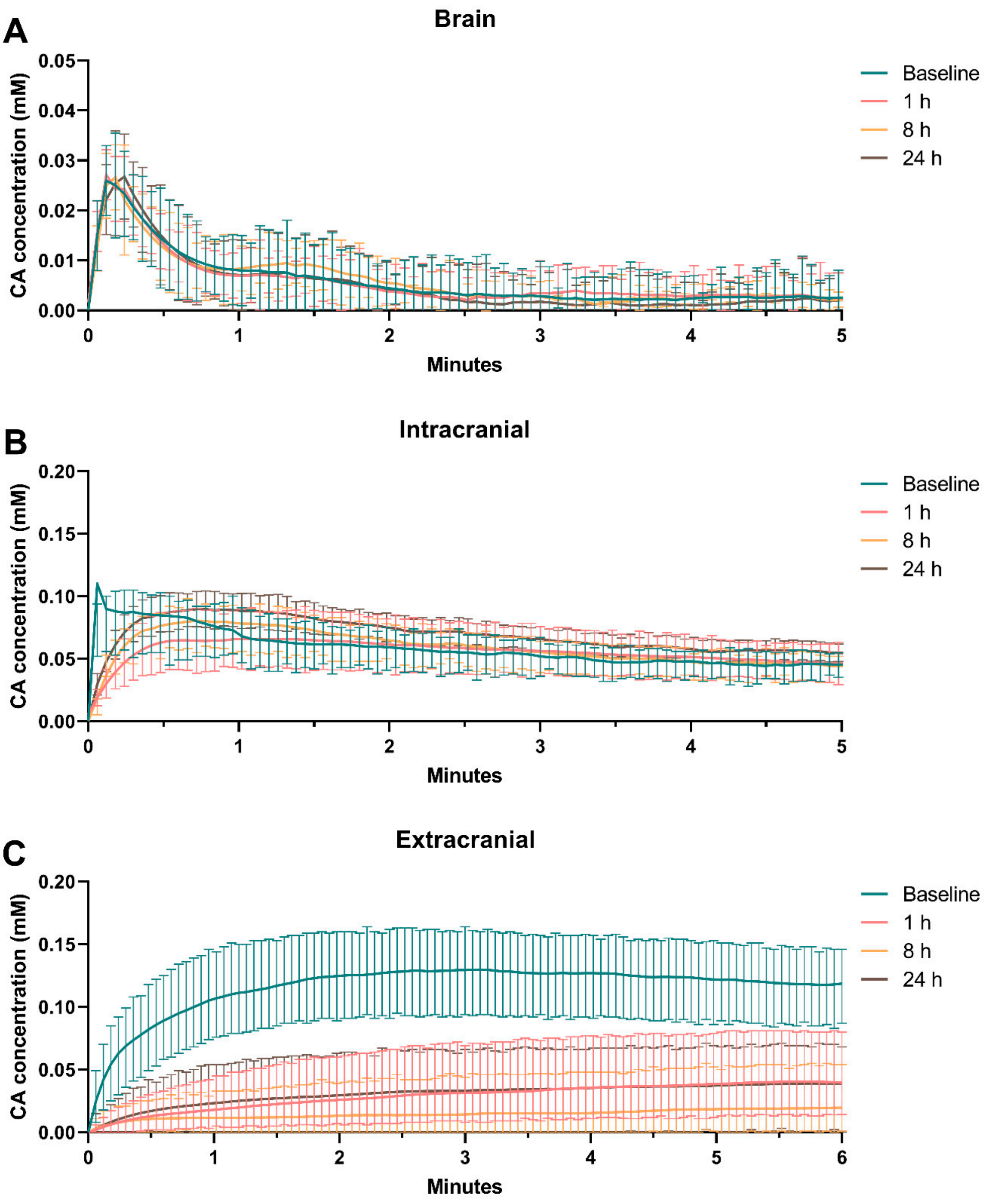

Figure S3. Contrast agent concentration curve of the brain (A), intracranial tumor (B) and extracranial tumor (C) before treatment, one hour, eight hours and 24 hours after treatment.

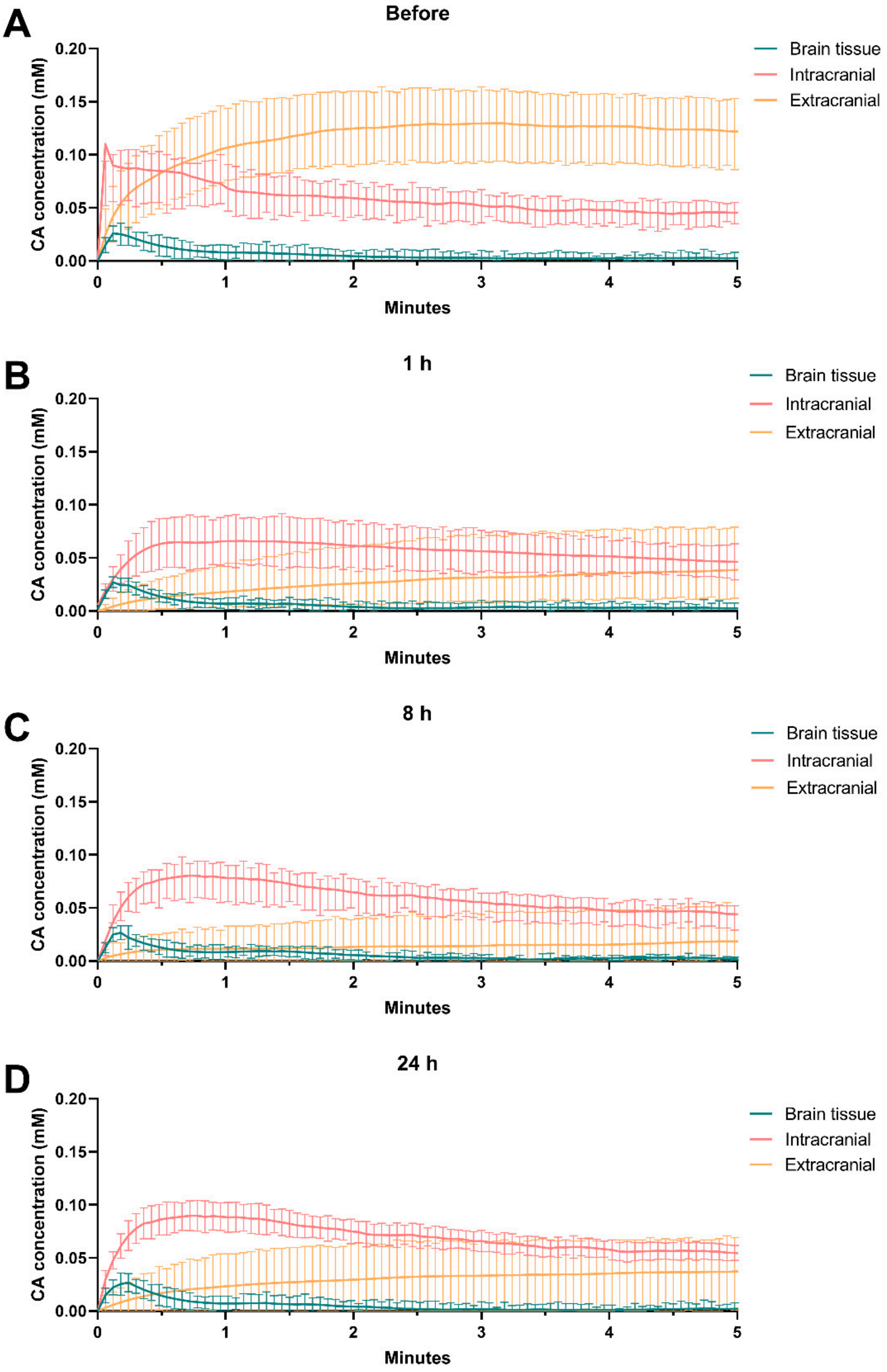

Figure S4. Contrast agent concentration curve by tumor site before treatment (A), one hour (B), eight hours (C) and 24 hours (D) after treatment.

File S1. Protocol for literature research.

The current supplementary file summarizes the literature search strategy on Web of Science.

- Search term "VDA": TS=(VDA);
- Search term "rodent": (((TS=(rat*)) OR TS=(rodent)) OR TS=(mice)) OR TS=(mouse);
- Search term "cancer": (((TS=(cancer*)) OR TS=(malignan*)) OR TS=(sarcoma)) OR TS=(neoplas*) or TS=(tumor*);
- Search term "brain": (((TS=(brain)) OR TS=(intracranial)) OR TS=(glio*)) OR TS=(cere*);
- Select original article: remove review, book section and conference articles.

Notes:

- CA4P/CA1P* include CA4P, CA1P, and their associated conjugate, derivative, or nanoparticles.
- For studies using two VDAs, they will be enrolled into both VDA groups.

File S2. Formulas for imaging analyses.

1. T2 mapping

The equation for standard T2 mapping is as follows:

$$SI_{(TE)} = S_0 * e^{-TE/T2}$$

where $SI_{(TE)}$ is the amplitude of the magnetization vector under pre-specified TE value, and S_0 the maximum magnitude.

2. T1 mapping

$$M_z(\Theta_n) = M_0 \frac{1 - e^{-\frac{TR}{T1}}}{1 - \cos(\Theta_n) e^{-\frac{TR}{T1}}} sin\Theta_n$$

where M_z is the longitudinal magnetization, M_0 is the magnetization at thermal equilibrium, TR is the pulse sequence repetition time, and Θ_n is the excitation flip angle.

3. Diffusion-weighted model

The analyses of diffusion-weighted Imaging (DWI) in our study consisted of methodologies: apparent diffusion coefficient (ADC). The equations for mono-exponential ADC is as follows respectively:

$$S_b = S_0 * e^{-b*ADC}$$

where S_b is the signal intensity for a particular b-value, S_0 is the signal intensity at b = 0 sec/mm^2.

4. Tissue concentration time curve (CTC) in dynamic contrast enhanced (DCE) model

The tissue concentration Ct of contrast agent (CA) during dynamic contrast enhanced perfusion experiment is solved as:

$$\frac{1}{T1_{(t)}} = \frac{1}{T1_0} + r1 * C_{t(t)}$$

where $T1_0$ is the T1 value before contrast injection, obtained from pre-contrast-enhanced T1 mapping, and r1 is the longitudinal relaxivity of applied CA. The T1 relaxation T1(t) after CA injection can be converted from the signal intensity (SI) time curve:

$$SI_{(t)} = S_0 * \frac{1 - e^{-TR/TI_{(t)}}}{1 - cos\alpha * e^{-TR/TI_{(t)}}} sin\alpha$$

in which S_0 is the longitudinal magnetization at equilibrium, derived from the Bloch equation of VIBE sequence, using signal measured from pre-contrast images:

$$SI_{pre} = S_0 * \frac{1 - e^{-TR/TI_0}}{1 - cos\alpha * e^{-TR/TI_0}} sin\alpha$$

and α is the flip angle in VIBE sequence.

5. DCE arterial input function (AIF) measurement:

The plasma concentration C_p of CA is determined by CA concentration in blood Cb:

$$C_p = \frac{C_b}{1 - Hct}$$

which is obtained from CTC of aorta, and the hematocrit H_{ct} is set to 42% in our study.
